# Receiving caregiver support and its association with hair hormones in people living with Alzheimer’s disease: The role of caregivers’ perspective taking

**DOI:** 10.1016/j.psyneuen.2026.107746

**Published:** 2026-01-10

**Authors:** Meng Huo, Kyungmin Kim, Casey K. Brown, Megan Gilligan, Wen Wang

**Affiliations:** a Department of Human Ecology, University of California, Davis, CA, USA; b Department of Child Development and Family Studies, College of Human Ecology, Seoul National University, Seoul, South Korea; c Integrated Major in Regional Studies and Spatial Analytics, Seoul National University, Seoul, South Korea; d Department of Psychology, Georgetown University, Washington, DC, USA; e Department of Human Development and Family Science, University of Missouri, Columbia, MO, USA; f Center for Translational Neuroscience, University of Oregon, Eugene, OR, USA

**Keywords:** Cortisol, Dehydroepiandrosterone (DHEA), Empathy, Caregiving

## Abstract

Receiving support in later life is often experienced as stressful, but for people living with dementia (PLWD) support is an unavoidable necessity for daily functioning. The current study examined the association between receiving support in this unique context and PLWD’s hair cortisol, dehydroepiandrosterone (DHEA), and DHEA-to-cortisol ratio, which serve as non-invasive, objective physiological measures that may reflect longer-term HPA-axis activity related to stress. Further, we explored whether caregivers’ perspective taking—their ability to understand PLWD’s thoughts and feelings—moderated associations between support receipt and hair hormones. Participants included 58 couples managing mild-to-moderate Alzheimer’s disease (*M*_age_ = 77.60 for PLWD; *M*_age_ = 75.48 for caregivers). PLWD self-reported the frequency of emotional and practical support received from their spousal caregivers. Hair samples were collected from the posterior vertex to assess cortisol and DHEA concentrations and were assayed using enzyme immunoassay (EIA) method. Caregivers reported their own perspective taking and both spouses’ demographic characteristics. Multiple regressions showed that receiving more frequent emotional support and less frequent practical support from spousal caregivers were associated with higher hair cortisol concentrations in PLWD. Yet, these associations were only evident if caregivers had greater perspective taking. In addition, caregiver perspective taking exacerbated the negative association between receiving emotional support and the DHEA-to-cortisol ratio. By using hair hormones, this study offers preliminary insights into PLWD’s stress-related physiological processes in the context of intensive caregiving. Findings refine our understanding of the benefits and costs of caregivers' perspective taking and inform caregiver interventions.

## 1. Introduction

Over 7 million Americans are living with Alzheimer’s disease and related dementias that disrupt their daily functioning and necessitate receipt of intensive support from their families, friends, and/or paid caregivers (Alzheimer’s Association, 2025). While support is essential, research has also linked receiving support to increased distress, anger, anxiety, depression, and even suicidal ideation (Brown and Vinokur, 2003; Lu and Argyle, 1992; Newsom and Schulz, 1998; Shrout et al., 2006). For people living with dementia (PLWD), this tension may be especially pronounced: although PLWD may benefit from support given their cognitive and functional deterioration, receiving support from caregivers can further undermine their already compromised sense of independence, eliciting chronic stress that in turn affects both partners’ health (de Boer et al., 2007; Feast et al., 2016; Livingston et al., 2024; van Gennip et al., 2016). Yet, it remains unclear how PLWD experience and react to caregivers’ support—whether it alleviates or contributes to their stress responses.

To address this gap, we examined how receiving practical and emotional support from caregivers was associated with PLWD’s hair hormones, which are not subject to social desirability and provide insights into physiological stress-related processes that are not captured by self-report measures (Qiao et al., 2017; Russell et al., 2012). When an individual is exposed to stress, their hypothalamic-pituitary-adrenal (HPA) axis is activated and releases *cortisol,* a glucocorticoid hormone, and *dehydroepiandrosterone* (DHEA), a hormone with anti-glucocorticoid properties (Kamin and Kertes, 2017). Cortisol helps mobilize resources throughout the body to respond to acute stress, but prolonged elevations are associated with neurotoxic effects (e.g., memory impairment; Dronse et al., 2023). DHEA has been shown to counteract some of the adverse effects and exert protective functions (Alhaj et al., 2006). Given the opposing physiological effects of these hormones, it is common to apply the DHEA-to-cortisol ratio to assess the balance between the two, with higher ratios indicating better physiological adaptation to stress (Lau et al., 2021). Hair hormones have been increasingly examined in gerontological studies as a promising non-invasive marker of long-term HPA-axis activity in older adults (Wright et al., 2015), even among those with dementia (Anderson et al., 2025; Kim et al., 2022). We extended prior work by examining how interpersonal processes such as receiving support might be associated with PLWD’s hair hormones.

Notably, PLWD’s reactions to receiving support are not uniform. Resistance to support can emerge in the context of difficult interpersonal dynamics (Allen and Wiles, 2013), and providing support effectively without eliciting resistance may depend on specific interpersonal characteristics (Howland et al., 2010; McGarry et al., 2021). One critical characteristic is perspective taking—the cognitive component of empathy focused specifically on the ability to understand PLWD’s thoughts and feelings (Davis, 2017). We asked whether caregivers’ perspective taking would moderate the association between receiving support and hair hormones in PLWD. Addressing this question can extend the caregiving literature and inform tailored interventions to mitigate stress that PLWD experience while receiving intensive care. In this study, we focused on couples including PLWD and spousal caregivers because spouses often co-reside with PLWD and provide intensive care.

We proposed a theoretical framework to guide our hypotheses, incorporating the Threat-to-Self-Esteem Model (Fisher et al., 1982; Nadler and Jeffrey, 1986), the Stress Process Model for PLWD (Judge et al., 2010), the contingent exchange perspective (Davey and Eggebeen, 1998), and the Russian Doll Model of empathy (de Waal, 2008). The Threat-to-Self-Esteem Model posits that receiving support may present a threat to one’s sense of independence and reduce their self-esteem, but how recipients react to the support varies depending on characteristics of the support recipient (e.g., perceived dependency), the support per se (e.g., the type of support), and the provider (e.g., how they help). The Stress Process Model (Judge et al., 2010) adds that perceived dependency (which could result from receiving support) can be a primary stressor for PLWD and lead to intrapsychic strains. However, receiving support may be necessary for PLWD as they incur progressive declines and become increasingly dependent on care.

How receiving support from caregivers affects PLWD may depend on the type of support received. According to the Threat-to-Self-Esteem Model (Fisher et al., 1982; Nadler and Jeffrey, 1986), emotional support reflects companionship and comfort and can be less threating to PLWD’s self-esteem, whereas practical support such as helping with errands, although necessary, may highlight PLWD’s incompetence to lead their lives independently and compromise their self-esteem. Yet, the contingent exchange perspective posits that the impact of receiving support may be contingent on specific situations and needs (Davey and Eggebeen, 1998). Compared to emotional support, practical support may be more essential to maintaining, if not promoting, PLWD’s quality of life. A daily study with older adults experiencing disabilities (which limit independent living in ways comparable to Alzheimer’s disease) found that older parents with disabilities reported more positive mood on days they received practical support from their adult children compared to days when they did not receive practical support (Huo et al., 2018). Compared to other disabilities, dementia involves cognitive impairments that often substantially undermine autonomy and increase reliance on others (van Gennip et al., 2016). Thus, we expected receiving more emotional support to be associated with lower hair cortisol, higher DHEA, and higher DHEA-to-cortisol ratio in PLWD. We did not specify the direction of the association for receiving practical support.

We then integrated the Russian Doll Model (de Waal, 2008; de Waal and Preston, 2017) to examine how these associations vary depending on caregivers’ perspective taking—a key trait that has been consistently linked to the motivation and delivery of support. This model proposes a layered structure of empathy, in which perspective taking lies at the outermost layer and represents the most cognitively demanding process to “stand in others’ shoes” and refine help based on cognitive awareness of others’ specific needs. That is, support may be more appropriate and effective when caregivers have high perspective taking (Devoldre et al., 2010; Huo et al., 2024; Verhofstadt et al., 2016). Most studies to date have focused on how caregivers’ perspective taking affects their own health and well-being (Huo et al., 2025), with little attention paid to its impact on PLWD’s experiences. One exception is a recent systematic review that links empathy to a variety of positive patient outcomes, such as better mental health (Nembhard et al., 2023). Yet, this review was limited to health care professionals and did not consider the multifaceted nature of empathy. Here, we expected caregivers’ perspective taking to moderate the association between receiving support and PLWD’s hair hormones in a positive manner.

In the current study, we examined how receiving support from caregivers was associated with PLWD’s hair cortisol, DHEA, and DHEA-to-cortisol ratio. Variation in older adults’ hair hormones is relatively understudied, such that prior research suggests mixed results on whether factors such as age, sex, physical health, and alcohol assumption consistently contribute to elevated hair cortisol (Feller et al., 2014; Lanfear et al., 2020). Here, we adjusted for characteristics that have been commonly examined in cortisol research (e.g., age, gender, education as an index for socioeconomic status/SES, physical health, medication use). For example, studies report mixed findings on whether older adults and women show higher or lower cortisol levels, but both groups consistently exhibit lower DHEA and lower DHEA-to-cortisol ratios (Stamou et al., 2023; Takeshita et al., 2025). Upper SES and healthier adults typically have lower cortisol levels, higher DHEA, and higher DHEA-to-cortisol ratios (Cohen et al., 2006). Medication use may either raise or suppress stress hormones depending on the specific pharmacological mechanism.

We tested the following hypotheses:

**Hypothesis 1**. Receiving emotional support more often would be associated with lower cortisol, higher DHEA, and higher DHEA-to-cortisol ratio in PLWD; there would be an association for receiving practical support but the direction was not specified.
**Hypothesis 2**. Caregivers’ perspective taking would moderate the associations between receiving support and PLWD’s hair hormones in a positive manner.

## 2. Material and methods

### 2.1. Sample

Data were from 58 couples in the *Dyadic Study on Empathy in Caregiving* (DSEC), which was conducted in the Northern California-Nevada area in 2022–2023 (Huo et al., 2024). The DSEC procedures were approved by the University of California, Davis (UCD) Institutional Review Board and recruitment primarily occurred via UCD Health and local support groups. This study was not pre-registered.

We screened participants to be community-dwelling, married or cohabiting couples in which one person was living with early-stage AD and the spouse self-identified as the primary caregiver providing the majority of assistance. Inclusion criteria for PLWD were (a) be age 55 and older and (b) have received a clinical diagnosis of mild or moderate (probable or possible) AD in their most recent doctor/neurologist visit. We did not exclude people who were diagnosed with mixed dementias (e.g., AD and vascular dementia), as long as they did not have frontotemporal dementia (FTD), which is known to compromise empathy to a greater extent than other dementias (Brown et al., 2020; Carr and Mendez, 2018; Fernandez-Duque et al., 2010). In addition to people with clinical diagnoses of AD, we included two people who had been diagnosed with MCI due to AD but had not followed up with their neurologists since then. Recruitment of people with moderate AD was decided on a case-by-case basis depending on whether PLWD could verbalize their thoughts and communicate; interviewers further excluded PLWD who were unable to comprehend study information during the consent or interview process. A sample of 72 couples were eligible (see a detailed sample description in Huo et al., 2024); PLWD and their spousal caregivers signed their own consent forms.

All 72 couples completed baseline interviews at home or in interview rooms on the UCD main campus and spouses were interviewed independently in separate rooms to rate their perspective taking, support exchanges and health. After the interviews, participants were invited to provide hair samples from the posterior vertex and trained interviewers collected hair of at least three centimeters long from the scalp. A total of 58 PLWD provided valid hair samples and had no missing data on key variables (perspective taking and support receipt) or covariates; their spousal caregivers all provided data on perspective taking. Additional components of the DSEC included semi-structured interviews and follow-up calls; data were not used in this study and thus not reported in detail.

### 2.2. Measures

#### 2.2.1. Frequency of support received from caregivers

PLWD reported how often they received emotional and practical support from their spousal caregivers, respectively, using items from the Intergenerational Support Scale (Fingerman et al., 2011). We provided examples for emotional support, such that “he/she (spouse) listens to your concerns or is available and provides comfort when you are upset.” We defined practical support as when “he/she (spouse) fixes something around the house, runs errands, or provides you a ride.” We modified the original 8-point scale (1 = *less than once a year or not at all* to 8 = *daily*) to 1 (*none or almost none of the time*), 2 (*a little of the time*), 3 (*a lot of the time*), and 4 (*all or nearly all of the time*).

#### 2.2.2. PLWD hair cortisol, DHEA, and DHEA-to-cortisol ratio

Hair samples were assayed in the Stress Physiology Integrative Team (SPIT) Laboratory at the University of Oregon following established protocols (Wang et al., 2019). Hair was segmented by 3 cm and washed twice in isopropanol using a rotator. After drying under forced air, 15 mg of clean hair was pulverized to fine powder using a ball mill. Cortisol and DHEA were extracted from the powdered hair using methanol for 24 h. Methanol was then evaporated under a stream of nitrogen gas, and the steroid extracts were reconstituted in assay diluent. Reconstituted samples were assayed immediately for cortisol and DHEA using commercially available enzyme immunoassay kit (Salimetrics, PA). To reduce the influence of extreme outliers (three outliers for PLWD cortisol and one outlier for PLWD DHEA), hormone concentrations were winsorized at the 95th percentiles prior to statistical analyses. Hormone concentrations were log-transformed prior to statistical analyses to normalize distributions, and the DHEA-to-cortisol ratio was calculated by dividing PLWD DHEA levels by cortisol levels. Higher DHEA-to-cortisol ratios are often used to reflect better physiological adaptation to stress (Lau et al., 2021).

#### 2.2.3. Caregivers' perspective taking

Caregivers self-rated perspective taking using the subscale of the Interpersonal Reactivity Index (IRI; Davis, 1983) modified to focus on perspective taking within couples (Péloquin and Lafontaine, 2010). We omitted three of the seven original items to reduce participant burden and replaced the word “partner” with specific spouse names to enhance clarity and ease of understanding (Huo et al., 2024). Caregivers indicated how well each item described them on a scale from 1 (*not at all*) to 5 (*a great deal*). An example item is “I try to look at [spouse name]’s side of a disagreement before I make a decision.” We calculated a mean score for across items to assess perspective taking (*α* =.73).

#### 2.2.4. Background characteristics as covariates

We adjusted for relevant background characteristics in our analyses. Caregivers reported demographic characteristics and health for PLWD: gender (1 = *male*, 0 = *female*), age (in years), education, physical health from 1 (*poor*) to 5 (*excellent*), and racial/ethnic groups. Education was measured on a scale of 1 (*less than high school*), 2 (*some high school*), 3 (*high school graduate*), 4 (*some college/vocation or trade school*), 5 (*college graduate*), and 6 (*post-college*). We recoded racial/ethnic minority status, such that PLWD identified as non-Hispanic White were non-minority and those who were of Hispanic origin or non-White were treated as racial/ethnic minority. PLWD self-reported whether they currently took any prescription or non-prescription medications on a regular basis (i.e., on daily basis for at least a month): 1 = *yes* and 0 = *no*.

### 2.3. Analytic strategy

As preliminary analyses, we ran descriptive statistics and bivariate correlations. To test our hypotheses, we ran multiple regressions in SPSS predicting hair cortisol, DHEA, and the DHEA-to-cortisol ration as separate outcomes. The frequencies of receiving emotional support and receiving practical support were entered into the models simultaneously as predictors, along with perspective taking and covariates (PLWD age, gender, education, self-rated health, medication use). When estimating moderation tests, we added interaction terms of support receipt × perspective taking into the models. All continuous variables were grand mean-centered. We explored significant interactions using simple slopes analyses to assess the associations between the frequency of support receipt and hair hormones at different levels of caregivers’ perspective taking (1 standard deviation above and below the mean; Huo et al., 2020).

## 3. Results

### 3.1. Sample characteristics

Table 1 describes the sample of 58 PLWD. On average, PLWD were 77.60 years old and mostly male (60 %). They had good physical health overall and received support from caregivers often. These PLWD’s caregivers were 75.48 years old and reported good to very good physical health. PLWD’s self-reports on the frequency of receiving emotional (*t* = 0.15, *p* = .88) and practical support (*t* = 0.96, *p* = .34) from caregivers did not differ from caregivers’ reports on the support they provided.

Bivariate correlations between key variables (see Table 2) revealed that the frequency of receiving emotional support was highly correlated with the frequency of receiving practical support, which was negatively associated with hair cortisol in PLWD. Caregivers’ perspective taking was positively associated with PLWD’s DHEA-to-cortisol ratio. None of the covariates were correlated with the hair hormones (outcomes of our analyses).

### 3.2. Hypothesis testing

We assessed the assumptions of linear regression (linearity, normality, homoscedasticity, and independence) and they were reasonably met across models. We present standardized coefficients below. Our first hypothesis focused on the associations of receiving emotional and practical support from caregivers with PLWD’s hair cortisol, DHEA, and DHEA-to-cortisol ratio. Multiple regressions revealed that receiving emotional support from spousal caregivers more often was associated with higher hair cortisol in PLWD (*β* = 0.47, *p* = .001), whereas receiving practical support more often was associated with lower hair cortisol (*β* = −0.55, *p* < .001). We did not observe any significant association involving PLWD’s DHEA or the DHEA-to-cortisol ratio. Caregiver perspective taking was associated with PLWD’s hair cortisol (*β* = −0. 27, *p* = .032) and DHEA-to-cortisol ratio (*β* = 0.33, *p* = .021), but it was not associated with PLWD’s DHEA (*β* = 0.09, *p* = .51). See Table 3.

We then tested our second hypotheses on the moderating effects of caregivers’ perspective taking and observed three effects on the association between receiving emotional support and PLWD’s hair cortisol (*β* = 0.56, *p* = .003), the association between receiving practical support and PLWD’s hair cortisol (*β* = −0.39, *p* = .013), and the association between receiving emotional support and PLWD’s DHEA-to-cortisol ratio (*β* = −0.41, *p* = .059; marginally significant).

**Fig. 1.**
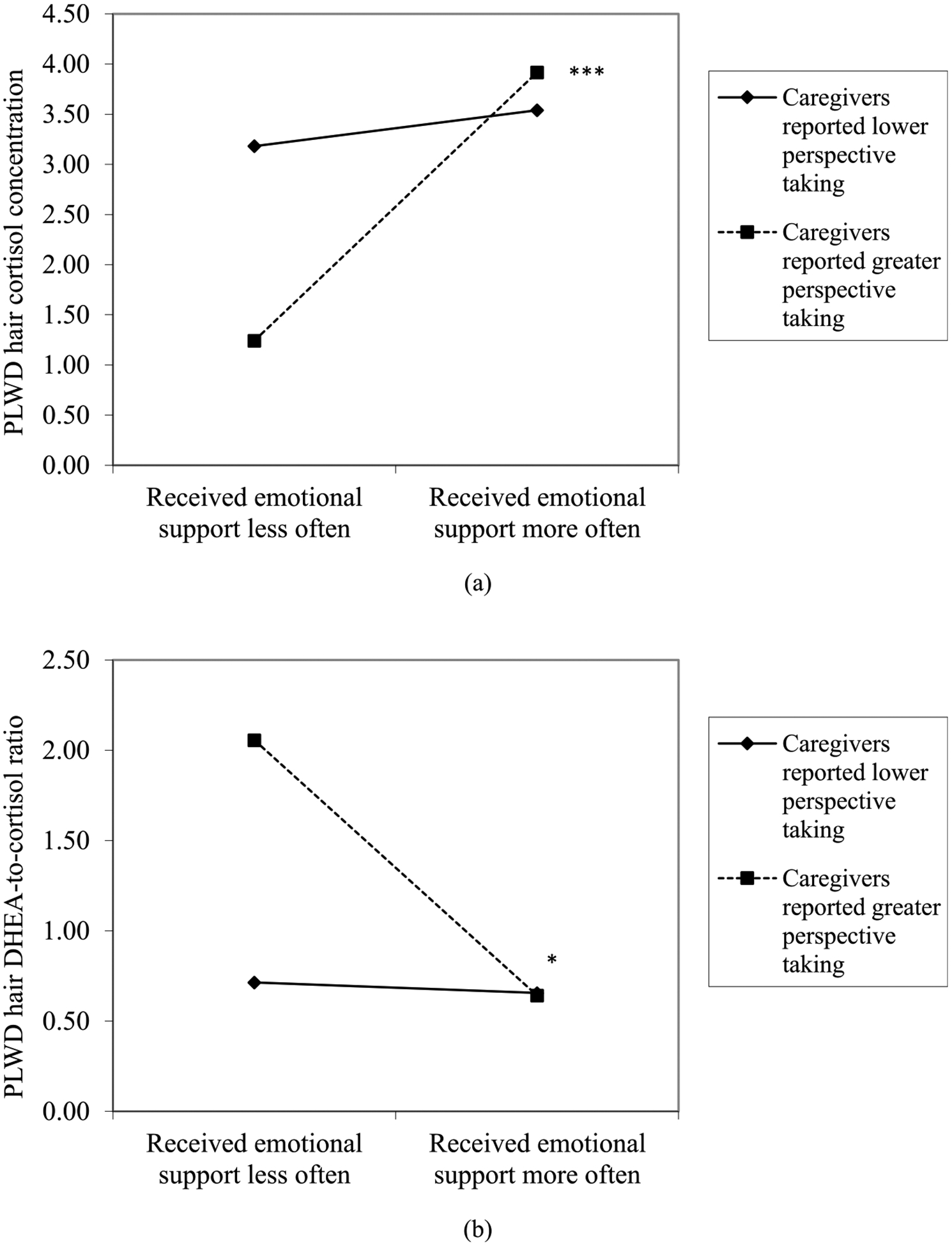
Interactions of Receiving Emotional Support × Caregiver Perspective Taking Predicting Hair Cortisol and DHEA-to-Cortisol Ratio in People Living With Dementia. Note. PLWD = person living with dementia. *p < .05. ***p < .001.

**Fig. 2.**
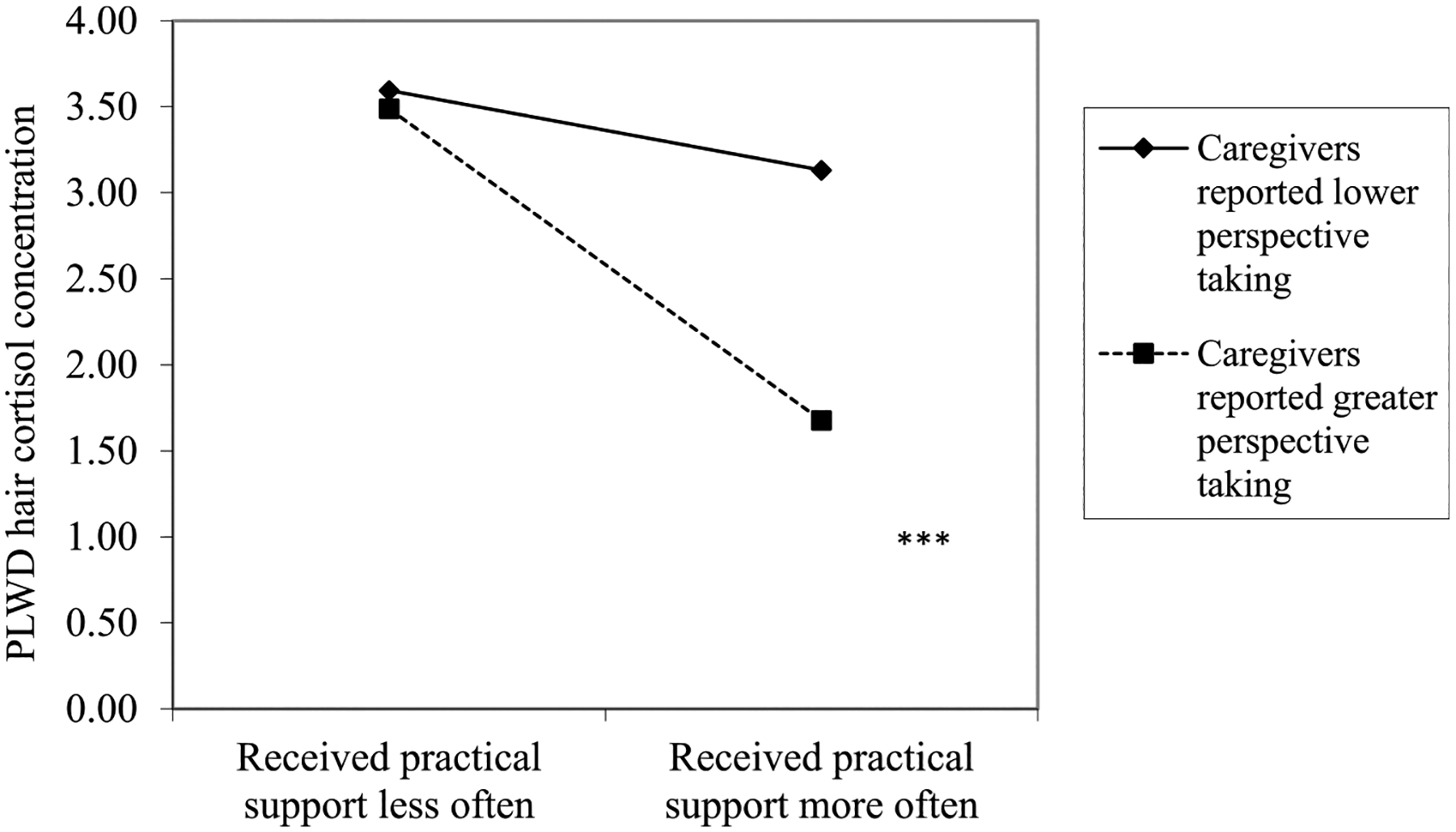
Interaction of Receiving Practical Support × Caregiver Perspective Taking Predicting Hair Cortisol in People Living With Dementia. Note. PLWD = person living with dementia. ***p < .001.

We estimated simple slopes analyses to interpret the moderation effects and found that all associations were evident when caregivers had high perspective taking but not when caregivers had low perspective taking. That is, only when PLWD had spousal caregivers whose reported high perspective taking, receiving emotional support from those caregivers more often was associated with higher hair cortisol (*t* = 4.31, *p* < .001) and lower DHEA-to-cortisol ratio (*t* = −2.55, *p* = .014; see Fig. 1a and Fig. 1b), whereas receiving practical support more often was associated with lower hair cortisol (*t* = −5.08, *p* < .001; see Fig. 2).

Post-hoc power analyses using G*Power indicated that the model for PLWD’s cortisol had sufficient power (1 - *β* =.96), whereas the models for DHEA and DHEA-to-cortisol were underpowered (1 - *β* =.24).

We re-estimated our analyses to examine emotional and practical support separately (see Supplementary Tables 1 and 2). Most findings reported above were non-significant, except that receiving practical support from caregivers more often was still associated with lower cortisol in PLWD. One new finding emerged: the association between receiving emotional support from spousal caregivers and PLWD’s DHEA was moderated by caregivers’ perspective taking (*β* = 0.35, *p* = .032) and only evident among those with greater perspective taking (*t* = 2.68, *p* = .010). We also re-estimated our analyses examining the average frequency of receiving emotional and practical support as the predictor and did not observe any significant results (see Supplementary Table 3).

## 4. Discussion

While receiving support has become an essential part of PLWD’s daily lives, we know little about how PLWD react to the support they receive. The current study addressed this gap by adopting established hair hormones to provide preliminary insights into PLWD’s physiological responses that manifest under the skin when they receive support from spousal caregivers. Hair measures capture hormone accumulation over weeks to months, offering a more integrated picture of HPA activation than repeated hormone assessments in saliva, urine, or blood (Wright et al., 2015). Notably, the type of support matters, in that receiving emotional support more often seems to co-occur with higher hair cortisol, whereas receiving more practical support correlates with lower hair cortisol. We observed individual variation in these associations by caregivers’ perspective taking, which adds to our understanding of the costs and benefits of perspective taking in the context of dementia caregiving.

We mostly observed significant results for hair cortisol and DHEA-to-cortisol ratio, which is consistent with prior work that considers these two biomarkers as more robust indicators of stress-related responses than DHEA (Pividori et al., 2024; Qiao et al., 2017). Examining these hair hormones is crucial as they have been associated with a variety of health and well-being outcomes. For example, a study with 1,876 healthy Irish older adults has found links between greater hair cortisol and poorer performance on memory and global cognitive tests (Feeney et al., 2020). In animal research, chronic stress as characterized by prolonged HPA axis activity and sustained hormone exposure has also long been shown to exacerbate the AD-related deficits and impairments (Alberini, 2009). Overall, this study adds to the growing body of work that has examined hair cortisol as an index of prolonged HPA axis activity in older adults (Lee et al., 2021; Pulopulos et al., 2014; Wright et al., 2015).

We expected (a) receiving support from caregivers to be associated with PLWD’s hair hormones and (b) caregivers’ perspective taking to moderate these associations. We found that receiving emotional support more often was associated with higher hair cortisol, whereas receiving practical support more often was associated with lower hair cortisol. However, both associations were significant only when caregivers had greater perspective taking but not when caregivers had low perspective taking.

The results regarding emotional support were contrary to our hypotheses, which predicted that (a) receiving emotional support would be associated with lower hair cortisol and (b) caregiver perspective taking would moderate this association in a positive manner. Emotional support, even if well-intentioned, does not always mitigate stress. We found evidence that when caregivers reported high perspective taking, receiving more emotional support from caregivers was associated with higher cortisol, lower DHEA, and lower DHEA-to-cortisol ratio, suggesting greater wear and tear in the body. Research on friendships has shown that perspective taking can increase distress by facilitating co-rumination—excessive discussion of problems between friends or dyad members (Smith and Rose, 2011). Caregivers’ attempting to take a partner’s perspective may elicit co-rumination about the frustration about the present and uncertainty about the future, which further exacerbates PLWD’s stress-related responses.

A caregiver with high perspective taking may be skilled at anticipating their PLWD’s emotional needs and reaching out readily whenever the PLWD feels upset. While PLWD may appreciate this kind of emotional comfort and companionship from spousal caregivers, they at times need space and do not feel comfortable with being too transparent about their emotional vulnerabilities. It is also possible that PLWD perceive receiving emotional support as a sign of weakness or excessive dependence. Consistent with this perspective, hair cortisol was the lowest and the DHEA-to-cortisol ratio was the highest when PLWD reported receiving less emotional support from caregivers high in perspective taking. This could occur because high-perspective-taking caregivers who offer subtle, “invisible” emotional support best meet the PLWD’s needs without threatening their sense of independence or self-esteem (Bolger et al., 2000). In other words, their support may be effective because it is minimally intrusive and goes unnoticed, rather than being delivered in a conventional, explicit way that undermines autonomy (Howland et al., 2010). However, we acknowledge the limitation of using cross-sectional data in this study; causal conclusions could not be drawn. For example, it is also possible that PLWD receive more emotional support when they exhibit greater physiological stress reactivity (as indexed by higher cortisol), particularly if their caregivers show a greater tendency to detect such changes.

Our finding of the inverse link between receiving practical support and hair cortisol was in line with the contingent exchange perspective (Davey and Eggebeen, 1998). According to this perspective, support that is matched to recipients’ needs and address their challenges can relieve stress most effectively. It also extends our prior work revealing how receiving practical support benefits the daily emotional well-being of older parents with disabilities (Huo et al., 2018). Receiving practical support may help PLWD’s manage limitations stemming from physical and cognitive declines and enhance their quality of life, especially when caregivers demonstrate high perspective taking. Indeed, perspective taking has been known to promote targeted helping that is tailored to the recipients’ specific needs (de Waal, 2008). According to optimal matching theory, such tailored support is more likely to align with PWLD’s needs, increasing their perception of partner responsiveness and effectiveness of support (Cutrona et al., 2007). Further, caregivers with greater perspective taking may provide support more strategically. Qualitative research suggests that respect from partners can help PLWD maintain their sense of personal dignity, despite gradually increasing reliance on others for assistance (van Gennip et al., 2016). Rather than blaming PLWD for forgetting something or redoing the laundry that has already been done, caregivers with high perspective taking may tend to provide gentle guidance and initiate joint problem solving; in these couples, PLWD reported feeling comfortable voicing their needs and assured that their needs would be addressed (Huo et al., 2025).

Several limitations to this study warrant consideration. The study sample is small (*N* = 58), but we extend the only two existing studies that assessed hair hormones in PLWD (*n* = 5 and 13, respectively; Anderson et al., 2025; Kim et al., 2022) and create a foundation for future studies. Some of our analyses are underpowered and multiple comparisons increase the risk of Type I errors. It is also noteworthy that some associations observed in the co-adjusted models (when receiving emotional and practical support were examined in the same models) did not remain significant when different types of support were assessed separately. Findings are preliminary and should be interpreted with caution, but this study provides an initial empirical step towards understanding how specific interpersonal processes may relate to PLWD’s hair hormones and prolonged HPA activation. We adjusted for relevant demographic characteristics, health, and medication use, and none was associated with hair hormones. Future research may explore other factors, particularly those specific to older age to better understand hair hormones in this life stage. Hair samples of three centimeters long allow us to assess hormones in the past 3 months, and we call for more research that collects multiple hair samples over time to potentially reveal more information about the trajectory of PLWD’s physiological responses.

## 5. Conclusion

We present the first study that incorporates hair hormones to understand PLWD’s physiological stress-related responses when receiving emotional and practical support and point out the costs and benefits of caregiver perspective taking across situations. This study advances our understanding of caregiving by adding PLWD’s perspectives and reveals individual variation in how receiving support from caregivers gets under the skin. Findings can inform caregiver empathy interventions that have primarily advocated for promoting perspective taking to protect caregivers’ health and well-being (Au et al., 2020; Jűtten et al., 2019). We provide empirical evidence that indicates how those interventions may potentially benefit PLWD but also call for more work aimed at optimizing the quality of care and reducing stress in PLWD.

## Supplementary Material

1

## Figures and Tables

**Table 1:** Sample Descriptive Characteristics (*N* = 58).

Variables	*M*	(*SD*)
**PLWD characteristics**		
Male, %	60	
Age	77.60	(6.26)
Education	5.12	(1.06)
Physical health	2.91	(1.08)
Medication use, %	81	
How often received emotional support	3.47	(0.82)
How often received practical support	3.57	(0.68)
Hair cortisol^a^	2.72	(1.05)
Hair DHEA^a^	2.40	(0.81)
Hair DHEA-to-cortisol ratio	1.05	(0.82)
**Caregiver perspective taking**	3.56	(0.80)

*Note*. PLWD = person living with dementia. DHEA = dehydroepiandrosterone.

a Hair biomarkers were winsorized and logged.

**Table 2:** Bivariate Correlations Among Study Variables (PLWD Characteristics).

Variables	1	2	3	4	5	6	7	8	9	10
1. Male	—									
2. Age	.04	—								
3. Education	.19	.07	—							
4. Physical health	.07	.19	.07	—						
5. Medication use	−.04	.01	−.16	.28*	—					
6. Received emotional support	−.10	−.00	−.15	.03	.16	—				
7. Received practical support	−.26	−.16	−.02	−.00	−.12	.37**	—			
8. Hair cortisol^a^	−.01	−.03	.05	−.11	.03	.15	−.33*	—		
9. Hair DHEA^a^	−.13	−.19	−.06	.02	.09	.20	.19	.08	—	
10. Hair DHEA-to-cortisol ratio	−.12	.07	.06	.16	.08	−.10	.06	−.55***	.35**	—

Note. PLWD = person living with dementia. DHEA = dehydroepiandrosterone.

a Hair biomarkers were winsorized and logged.

* *p* < .05.

** *p* < .01.

*** *p* < .001.

**Table 3:** Multiple Regressions Predicting Hair Hormones in People Living With Dementia.

	PLWD hair cortisol		PLWD hair DHEA		PLWD DHEA-to-cortisol ratio	
Variables	Main effects	Moderation effects	Main effects	Moderation effects	Main effects	Moderation effects
Received emotional support	0.47**	0.73***	0.16	0.32	−0.25	−0.45*
× Caregiver perspective taking	—	0.56**	—	0.30	—	−0.41
Received practical support	−0.55***	−0.55***	0.09	0.04	0.14	0.15
× Caregiver perspective taking	—	−0.39*	—	0.06	—	0.28
Caregiver perspective taking	−0.27*	−0.38**	0.09	0.10	0.33*	0.40**
**Covariates**						
PLWD male	−0.13	−0.16	−0.06	−0.09	−0.09	−0.07
PLWD age	−0.21	−0.32*	−0.29	−0.30*	0.09	0.17
PLWD education	0.17	0.10	0.03	−0.02	0.04	0.09
PLWD physical health	−0.02	0.03	0.14	0.18	0.13	0.10
PLWD medication use	−0.11	−0.06	0.05	0.10	0.14	0.10

*Note*. PLWD = person living with dementia. DHEA = dehydroepiandrosterone. Standardized coefficients (*β*) were presented in table.

* *p* < .05.

** *p* < .01.

*** *p* < .001.

## Data Availability

All de-identified data, analytic methods, and study materials would be available to other researchers for replication purposes. Please contact the corresponding author for details. Research reported in this manuscript was not pre-registered.

## Appendix A. Supporting information

Supplementary data associated with this article can be found in the online version at doi:10.1016/j.psyneuen.2026.107746.
