# Supplementary material for: Receiving caregiver support and its association with hair hormones in people living with Alzheimer’s disease: The role of caregivers’ perspective taking
Source: Psychoneuroendocrinology. Author manuscript; Available in PMC 2026 Jul 1. (PMC13320609; doi:10.1016/j.psyneuen.2026.107746)
Supplement: 1 [file NIHMS2189673-supplement-1.docx]

**Supplementary Table 1**

*Multiple Regressions Predicting Hair Hormones in People Living With Dementia From Receiving Emotional Support*

|  | **PLWD hair cortisol** | | |  | **PLWD hair DHEA** | | |  | **PLWD DHEA-to-cortisol ratio** | | |
| --- | --- | --- | --- | --- | --- | --- | --- | --- | --- | --- | --- |
| Variables | Main effects |  | Moderation effects |  | Main effects |  | Moderation effects |  | Main effects |  | Moderation effects |
| Received emotional support | 0.25 |  | 0.32 |  | 0.20 |  | 0.36* |  | -0.19 |  | -0.27 |
| × Caregiver perspective taking | — |  | 0.16 |  | — |  | 0.35* |  | — |  | -0.17 |
| Caregiver perspective taking | -0.23 |  | -0.23 |  | 0.08 |  | 0.08 |  | 0.32* |  | 0.32* |
| **Covariates** |  |  |  |  |  |  |  |  |  |  |  |
| PLWD male | 0.00 |  | -0.01 |  | -0.08 |  | -0.10 |  | -0.13 |  | -0.12 |
| PLWD age | -0.12 |  | -0.13 |  | -0.30 |  | -0.32* |  | 0.07 |  | 0.08 |
| PLWD education | 0.14 |  | 0.12 |  | 0.03 |  | -0.02 |  | 0.04 |  | 0.07 |
| PLWD physical health | -0.06 |  | -0.05 |  | 0.15 |  | 0.18 |  | 0.14 |  | 0.13 |
| PLWD medication use | 0.01 |  | 0.05 |  | 0.03 |  | 0.10 |  | 0.10 |  | 0.07 |

*Note*. PLWD = person living with dementia. DHEA = dehydroepiandrosterone. Standardized coefficients (*β*) were presented in table.

**p* < .05. ***p* < .01. ****p* < .001.

**Supplementary Table 2**

*Multiple Regressions Predicting Hair Hormones in People Living With Dementia From Receiving Practical Support*

|  | **PLWD hair cortisol** | | |  | **PLWD hair DHEA** | | |  | **PLWD DHEA-to-cortisol ratio** | | |
| --- | --- | --- | --- | --- | --- | --- | --- | --- | --- | --- | --- |
| Variables | Main effects |  | Moderation effects |  | Main effects |  | Moderation effects |  | Main effects |  | Moderation effects |
| Received practical support | -0.36* |  | -0.34* |  | 0.16 |  | 0.12 |  | 0.04 |  | 0.03 |
| × Caregiver perspective taking | — |  | -0.10 |  | — |  | 0.22 |  | — |  | 0.28 |
| Caregiver perspective taking | -0.17 |  | -0.19 |  | 0.13 |  | 0.18 |  | 0.27* |  | 0.29* |
| **Covariates** |  |  |  |  |  |  |  |  |  |  |  |
| PLWD male | -0.11 |  | -0.10 |  | -0.05 |  | -0.07 |  | -0.10 |  | -0.11 |
| PLWD age | -0.15 |  | -0.17 |  | -0.27 |  | -0.23 |  | 0.06 |  | 0.07 |
| PLWD education | 0.11 |  | 0.11 |  | 0.01 |  | -0.00 |  | 0.07 |  | 0.07 |
| PLWD physical health | -0.05 |  | -0.05 |  | 0.13 |  | 0.14 |  | 0.15 |  | 0.15 |
| PLWD medication use | 0.00 |  | -0.01 |  | 0.08 |  | 0.11 |  | 0.08 |  | 0.09 |

*Note*. PLWD = person living with dementia. DHEA = dehydroepiandrosterone. Standardized coefficients (*β*) were presented in table.

**p* < .05. ***p* < .01. ****p* < .001.

**Supplementary Table 3**

*Multiple Regressions Predicting Hair Hormones in People Living With Dementia From Receiving Support (Averaged Across Types)*

|  | **PLWD hair cortisol** | | |  | **PLWD hair DHEA** | | |  | **PLWD DHEA-to-cortisol ratio** | | |
| --- | --- | --- | --- | --- | --- | --- | --- | --- | --- | --- | --- |
| Variables | Main effects |  | Moderation effects |  | Main effects |  | Moderation effects |  | Main effects |  | Moderation effects |
| Received support | -0.03 |  | -0.04 |  | 0.21 |  | 0.24 |  | -0.10 |  | -0.10 |
| × Caregiver perspective taking | — |  | -0.10 |  | — |  | 0.26 |  | — |  | 0.01 |
| Caregiver perspective taking | -0.17 |  | -0.18 |  | 0.10 |  | 0.14 |  | 0.29* |  | 0.29* |
| **Covariates** |  |  |  |  |  |  |  |  |  |  |  |
| PLWD male | -0.02 |  | -0.01 |  | -0.05 |  | -0.07 |  | -0.13 |  | -0.14 |
| PLWD age | -0.10 |  | -0.11 |  | -0.28* |  | -0.26 |  | 0.05 |  | 0.05 |
| PLWD education | 0.11 |  | 0.12 |  | 0.03 |  | -0.01 |  | 0.06 |  | 0.06 |
| PLWD physical health | -0.07 |  | -0.08 |  | 0.14 |  | 0.15 |  | 0.15 |  | 0.15 |
| PLWD medication use | 0.06 |  | 0.03 |  | 0.05 |  | 0.12 |  | 0.07 |  | 0.08 |

*Note*. PLWD = person living with dementia. DHEA = dehydroepiandrosterone. Standardized coefficients (*β*) were presented in table.

**p* < .05. ***p* < .01. ****p* < .001.
